# Identification of Mast Cell-Based Molecular Subtypes and a Predictive Signature in Clear Cell Renal Cell Carcinoma

**DOI:** 10.3389/fmolb.2021.719982

**Published:** 2021-09-27

**Authors:** Hanxiang Liu, Yi Yang

**Affiliations:** Pediatric Urology, Shengjing Hospital of China Medical University, Shenyang, China

**Keywords:** renal clear cell carcinoma, mast cell, WGCNA, TCGA, arrayexpress, ICGC, clinical prognostic model

## Abstract

**Background:** Kidney renal clear cell carcinoma (KIRC) is a common malignant tumor of the urinary system. Surgery is the preferred treatment option; however, the rate of distant metastasis is high. Mast cells in the tumor microenvironment promote or inhibit tumorigenesis depending on the cancer type; however, their role in KIRC is not well-established. Here, we used a bioinformatics approach to evaluate the roles of mast cells in KIRC.

**Methods:** To quantify mast cell abundance based on gene sets, a single-sample gene set enrichment analysis (ssGSEA) was utilized to analyze three datasets. Weighted correlation network analysis (WGCNA) was used to identify the genes most closely related to mast cells. To identify new molecular subtypes, the nonnegative matrix factorization algorithm was used. GSEA and least absolute shrinkage and selection operator (LASSO) Cox regression were used to identify genes with high prognostic value. A multivariate Cox regression analysis was performed to establish a prognostic model based on mast cell-related genes. Promoter methylation levels of mast cell-related genes and relationships between gene expression and survival were evaluated using the UALCAN and GEPIA databases.

**Results:** A prolonged survival in KIRC was associated with a high mast cell abundance. KIRC was divided into two molecular subtypes (cluster 1 and cluster 2) based on mast cell-related genes. Genes in Cluster 1 were enriched for various functions related to cancer development, such as the TGFβ signaling pathway, renal cell carcinoma, and mTOR signaling pathway. Based on drug sensitivity predictions, sensitivity to doxorubicin was higher for cluster 2 than for cluster 1. By a multivariate Cox analysis, we established a clinical prognostic model based on eight mast cell-related genes.

**Conclusion:** We identified eight mast cell-related genes and constructed a clinical prognostic model. These results improve our understanding of the roles of mast cells in KIRC and may contribute to personalized medicine.

## Introduction

Clear cell renal cell carcinoma (KIRC) accounts for approximately 65–70% of all renal cell carcinomas (Warren and Harrison, 2018; Siegel et al., 2020). Metastasis is the main cause of death in patients with KIRC (Li et al., 2018). The early clinical features are not obvious and are difficult to identify. Therefore, some patients with KIRC have metastases when they are first diagnosed (Motzer et al., 1996). Although surgical treatment achieves good results, the 5-years survival rate for patients with metastatic KIRC is still low (Heidenreich et al., 2012; Sara et al., 2016). Studies on immune checkpoint inhibitors have made significant advances for KIRC treatment; however, the response to immunotherapy in patients with KIRC varies greatly across individuals (Fang et al., 2020). Therefore, it is necessary to identify therapeutic targets and effective predictors for early diagnosis and treatment.

Mast cells are one of the main components of the tumor immune microenvironment. The mast cell density is elevated in various types of tumors (Marone et al., 2016). Mast cells can be attracted by chemotactic molecules produced by tumor cells, thus producing a variety of angiogenic and lymphangiogenic factors, thereby contributing to tumor growth and metastasis (Boesiger et al., 1998; Abdel-Majid and Marshall, 2004; Taskinen et al., 2008; Detoraki et al., 2010; Melillo et al., 2010; Theoharides et al., 2010; Sismanopoulos et al., 2012). While many studies have demonstrated that mast cells can promote tumor development, others have shown that mast cells have tumor-inhibitory effects (Dabiri et al., 2004; Amini et al., 2007). A poor prognosis in KIRC has been linked to the existence of mast cells (Hiroshi et al., 1999; Melillo et al., 2010; Strouch et al., 2010; David et al., 2011; Rao et al., 2016). Previous studies have shown that mast cells can be used as targets for immunotherapy of solid tumors (Oldford and Marshall, 2015). Beuselinck et al. used unsupervised transcriptome analysis to identify four robust KIRC subtypes that were associated with different responses to sunitinib treatment (Beuselinck et al., 2015). Zhao et al. classified KIRC in the Chinese population into three classes based on gene expression, which provides practical guidelines on clinical treatment of patients with KIRC (Zhao et al., 2020). However, few studies have examined the role of mast cells in KIRC and the molecular mechanisms underlying their effects.

In this study, we used a bioinformatics approach to evaluate the prognostic value of mast cell-related genes in KIRC. In particular, we used a single-sample gene set enrichment analysis (ssGSEA) to quantify mast cell abundance in three KIRC datasets. Then, a series of statistical analyses, including a univariate Cox regression analysis, weighted correlation network analysis (WGCNA), GSEA, Least Absolute Shrinkage and Selection Operator (LASSO) Cox analysis, and Kaplan–Meier survival analysis, were performed to identify mast cell-related genes that may regulate the development of KIRC and to develop clinical prognostic models. These results will improve our understanding of the role of mast cells in KIRC and provide a basis for personalized treatment.

## Methods

### Processing of KIRC Patient Data Set

Clinical information and KIRC transcriptome sequencing data were downloaded from The Cancer Genome Atlas (TCGA, https://portal.gdc.cancer.gov/repository), including data for 539 KIRC and 72 normal cases. The E-MTAB-1980 dataset (*n* = 101) was downloaded from the ArrayExpress database (https://www.ebi.ac.uk/arrayexpress/). Similarly, sample information for KIRC (*n* = 91) was downloaded from the International Cancer Genome Consortium (ICGC) database (https://dcc.icgc.org/). Immune-related genes were derived from the Immunology database and Analysis Portal (ImmPort) database (https://www.immport.org/home). A mast cell gene set (Supplementary Table S1) was obtained from a previous study (Bindea et al., 2013).

### Quantification of Mast Cell Abundance

Mast cell abundance was quantified in three bladder datasets using ssGSEA based on the mast cell gene set using the GSVA R package (Hänzelmann et al., 2013).

### Identification of Mast Cell-Related Genes and Molecular Subtypes

WGCNA was performed using the R package “WGCNA” (Langfelder and Horvath, 2008) to identify highly correlated gene modules among samples and these modules were used for subsequent analyses. WGCNA was based on 1670 immune-related genes from TCGA-KIRC, and the relationships between single genes and mast cell density were quantified by gene significance. Module membership was evaluated as the correlation between the gene expression profiles and module characteristic genes. The total number of non-gray modules was eight. The brown module was most highly correlated with the mast cell density (*r* = 0.58, p = 9e-46). This module contained 258 mast cell-related genes. Among the 258 mast cell-related genes, 250 were consistently found in all ArrayExpress and the International Cancer Genome Consortium (ICGC) datasets and were used for an non-negative matrix factorization (NMF) clustering analysis. Gene Ontology (GO) and Kyoto Encyclopedia of Genes and Genomes (KEGG) pathway enrichment analyses of the 250 genes were performed using the clusterProfiler package in R (Yu et al., 2012). We identified functional pathways related to cluster 1 in TCGA dataset and used h.all.v7.1.symbols.gmt as the reference gene set for GSEA. The analysis was performed using 1000 permutations with a <0.05 false discovery rate (FDR) as the screening threshold, and GSEA version 4.0.1. ESTIMATE (Yoshihara et al., 2013) and CIBERSORT (Newman et al., 2019) algorithms were used to explore the relationship between molecular subtypes and tumor immune microenvironment.

### Chemotherapeutic Response and Immunotherapeutic Response Prediction

The responses to doxorubicin and sunitinib, two commonly used chemotherapeutic drugs, were predicted for each sample according to the Genomics of Drug Sensitivity in Cancer (GDSC, https://www.cancerrxgene.org/) using the R package “pRRophetic.” Based on 10-fold cross-validation of the GDSC training set, the prediction accuracy was evaluated, and ridge regression was used to estimate the IC_50_ values for the samples. Repeated gene expression estimates were summarized as an average value, and default values were used for all parameters setting the tissue type to “allSolidTumours” and using “combat” for batch effect removal (Geeleher et al., 2014). All parameters were set to the default values. We then compared the TCGA KIRC expression profile of cluster 1 and 2 with another published dataset that contained the data of 47 patients with melanoma who responded to immunotherapies using subclass mapping method (https://cloud.genepattern.org/gp) (Lu et al., 2019).

### Construction and Verification of Clinical Prognostic Model Based on Mast Cell-Related Genes

To identify the mast cell-related genes most closely related to prognosis, a Cox regression analysis with the LASSO penalty was performed using the R package “glmnet.” To construct the optimal clinical prognostic model of mast cell-related genes, a multiple regression analysis was used. We developed the formula for the risk score as follows: Risk Score = coef (gene 1) × expr (gene 1) + coef (gene 2) × expr (gene 2) + coef (gene 3) × expr (gene 3) + …. + coef (gene N) × expr (gene N). Patients were classified into a high-risk and low-risk group according to the median value of the risk scores of all samples in each dataset.

### Survival Analysis and Methylation Analysis

To evaluate the relationship between survival and the expression of eight genes in the model, the GEPIA database (http://gepia.cancer-pku.cn/) was used (Tang et al., 2017). The UALCAN database (http://ualcan.path.uab.edu/) was used to analyze the promoter methylation levels of genes used to construct a clinical prognostic model (Chandrashekar et al., 2017).

### Statistical Analyses

Differences in overall survival (OS) among groups were compared using the Kaplan-Meier analysis and log-rank test. A multi-time receiver operating characteristic (ROC) analysis and area under the curve (AUC) were used to evaluate signal specificity and sensitivity. R (version 4.0.2) was used for all statistical analyses. Statistical significance was set at *p* < 0.05.

## Results

### Mast Cell Abundance is Beneficial for Survival of Patients With KIRC

**FIGURE 1:**
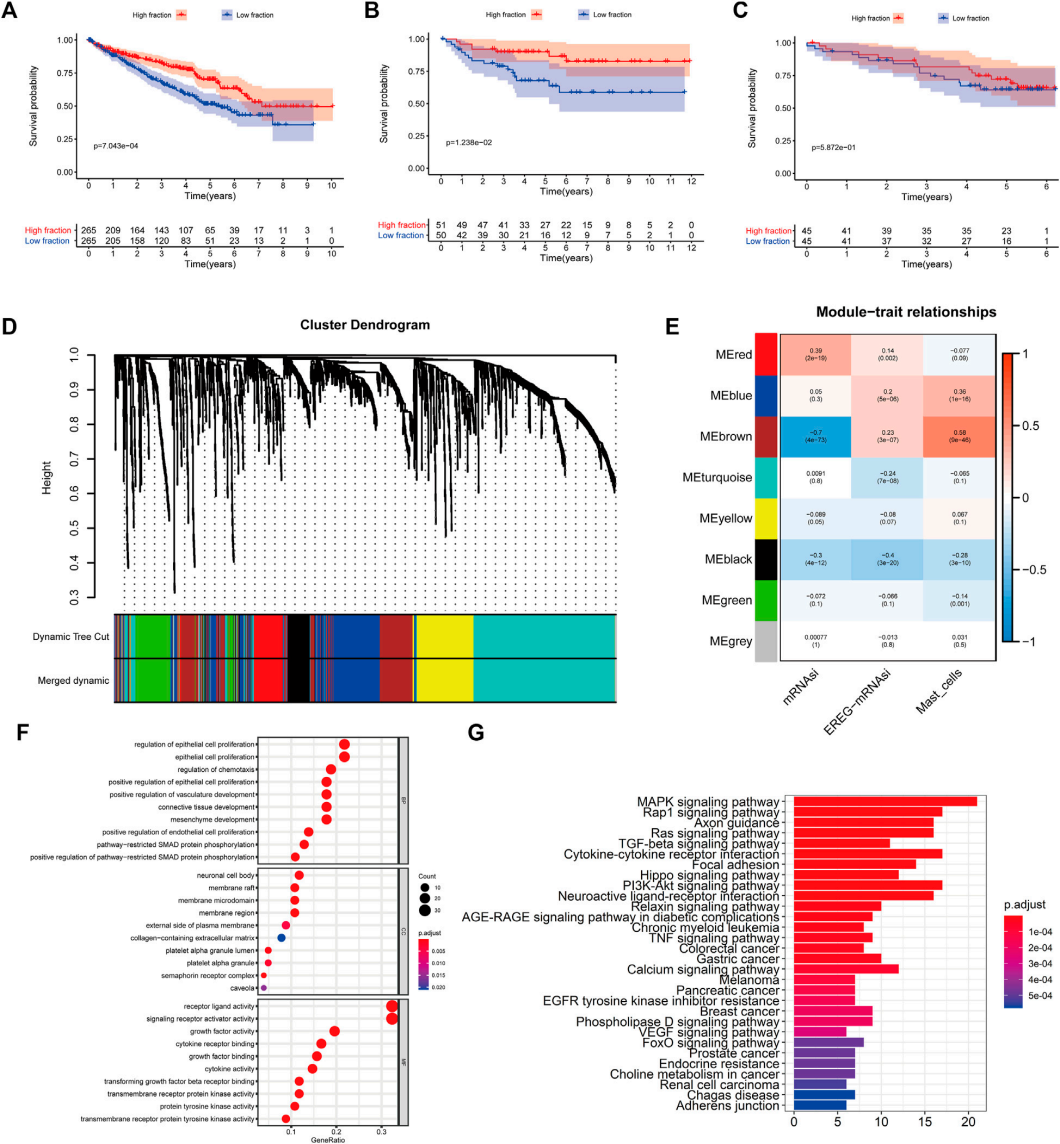
**(A–C)** Kaplan–Meier curves for patients with bladder cancer (BLCA) showed that in the six cohorts, patients with a low fibroblast abundance have a better prognosis than that of patients with a high fibroblast abundance [**(A)**: The Cancer Genome Atlas (TCGA); **(B)**: E-MTAB-1980; **(C)**: International Cancer Genome Consortium (ICGC)] **(C)** Using weighted correlation network analysis (WGCNA), eight modules were identified. **(D)** The brown module was most highly correlated with mast cells (cor: 0.58, p = 9e-46). **(E, F)** Functional enrichment analysis of 258 mast cell-related genes.

To quantify mast cell abundance based on a mast cell gene set in three KIRC datasets (TCGA, ArrayExpress, and ICGC cohorts), ssGSEA was used. A univariate Cox analysis showed that the mast cell gene set was a protective factor for KIRC (Table 1). In addition, we divided the samples in the three data sets into low abundance and high abundance groups based on ssGSEA scores. A high abundance of mast cells was beneficial for the survival of patients with KIRC (Figures 1A–C).

**TABLE 1:** Univariate Cox regression analysis of fibroblast abundance in The Cancer Genome Atlas (TCGA), E-MTAB-1980, and International Cancer Genome Consortium (ICGC) cohorts.

Datasets	HR	HR.95 L	HR.95H	*p*-value
TCGA	0.004201883	0.000320462	0.055094827	3.08E-05
E-MTAB-1980	4.95E-05	2.81E-08	0.087285488	0.009339429
ICGC	0.164918533	0.000142324	191.0997492	0.616586053

### Identification of Mast Cell-Related Genes and Molecular Subtypes

**FIGURE 2:**
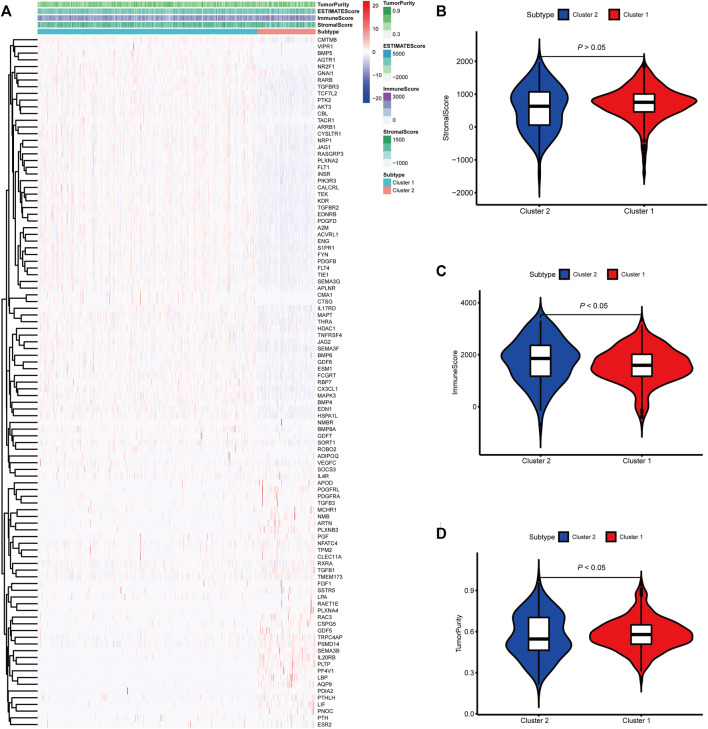
Molecular subtypes identified based on mast cell-related genes in The Cancer Genome Atlas (TCGA) cohort. **(A)** Heatmap of differences between cluster 1 and cluster 2. **(B–D)** Differential analyses of the immune score, stromal score, and tumor purity between cluster 1 and cluster 2.

**FIGURE 3:**
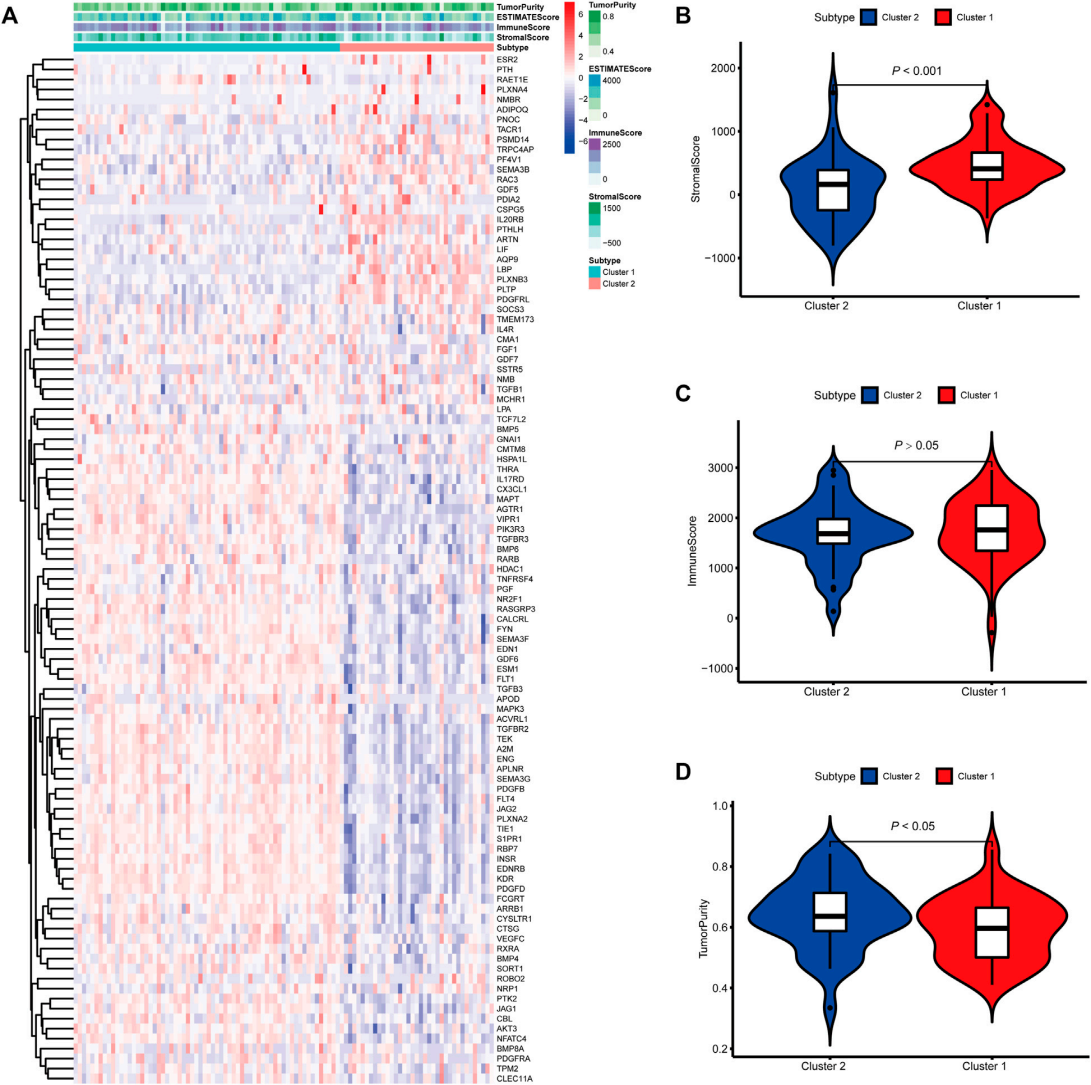
Molecular subtypes identified based on mast cell-related genes in the E-MTAB-1980 cohort. **(A)** Heatmap of differences between cluster 1 and cluster 2. **(B–D)** Differential analyses of the immune score, stromal score, and tumor purity between cluster 1 and cluster 2.

**FIGURE 4:**
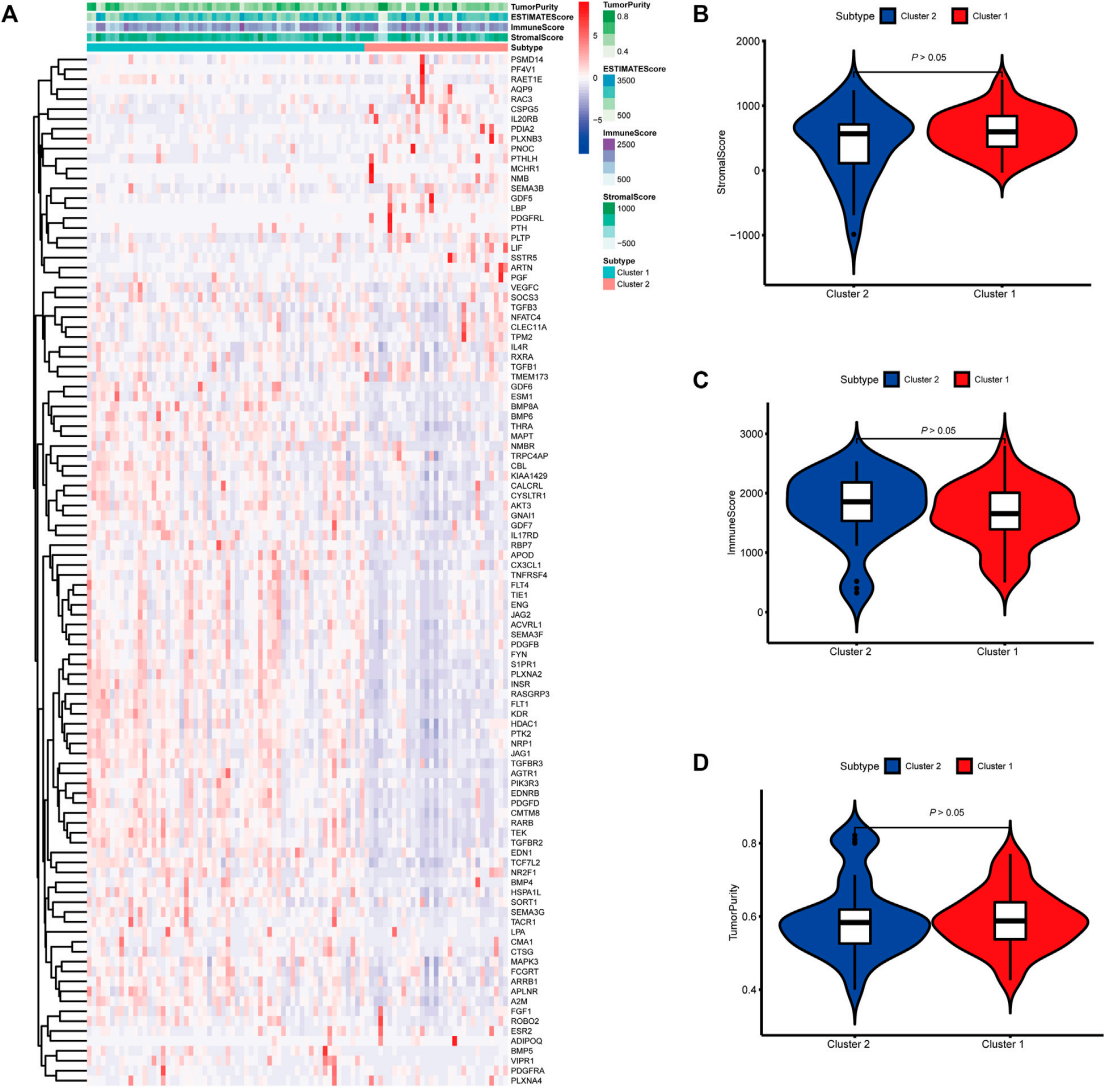
Molecular subtypes identified based on mast cell-related genes in the International Cancer Genome Consortium (ICGC) cohort. **(A)** Heatmap of differences between cluster 1 and cluster 2. **(B–D)** Differential analyses of the immune score, stromal score, and tumor purity between cluster 1 and cluster 2.

**FIGURE 5:**
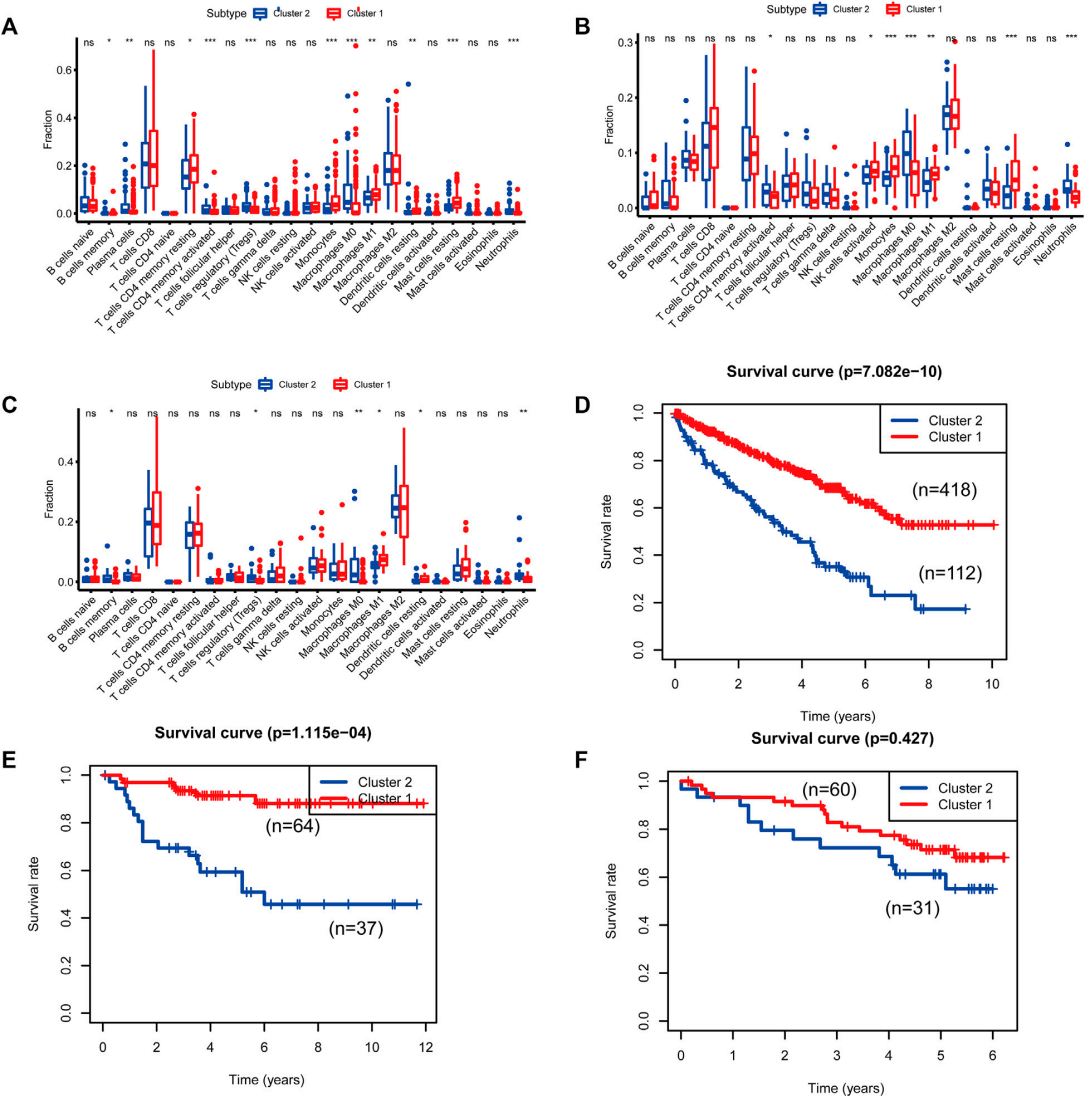
Differences in immune cell populations and survival between the two molecular subtypes of cluster 1 and cluster 2. [**(A, D)**: The Cancer Genome Atlas (TCGA); **(B, E)**: E-MTAB-1980; **(C, F)**: International Cancer Genome Consortium (ICGC)].

**FIGURE 6:**
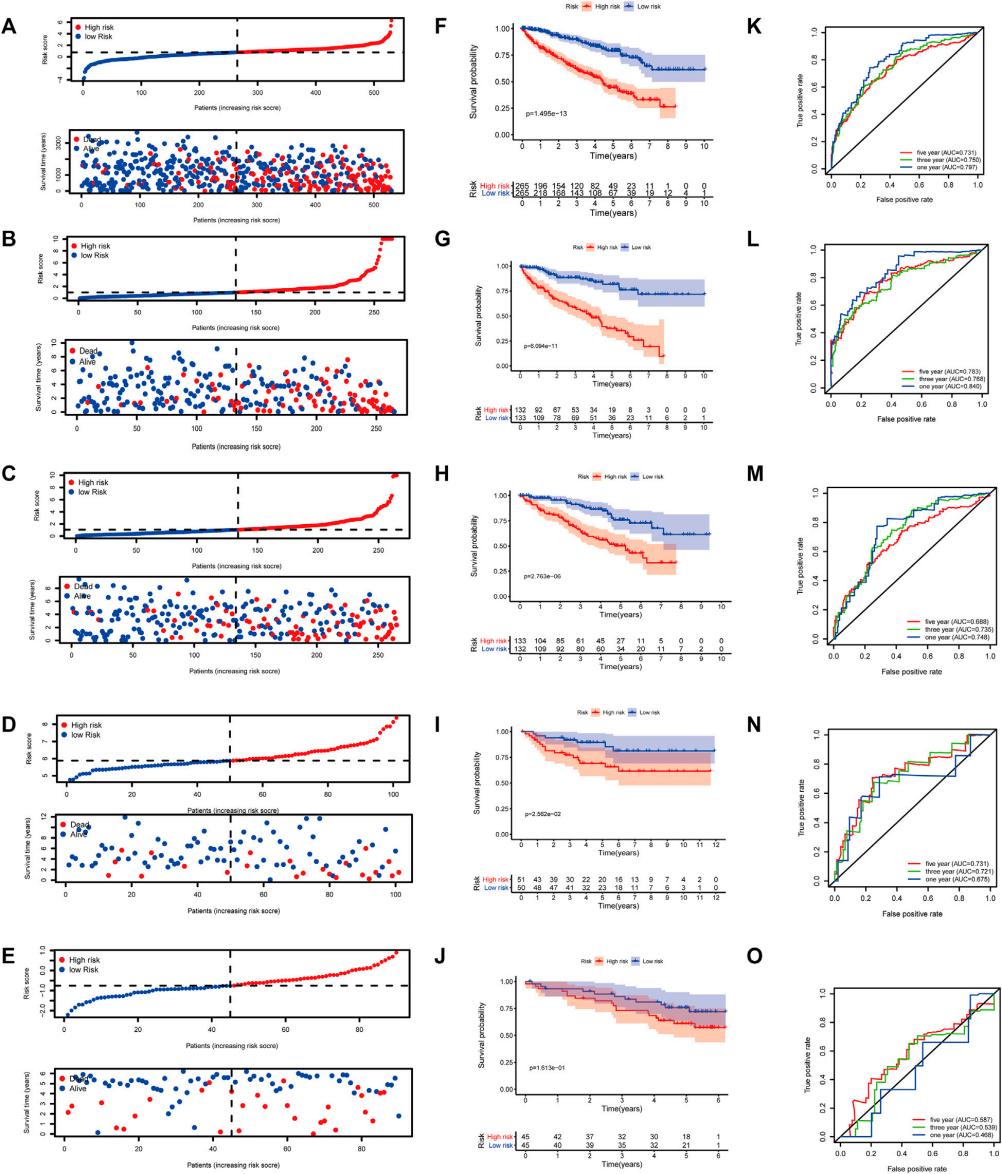
Gene set enrichment analysis (GSEA) of Kyoto Encyclopedia of Genes and Genomes (KEGG) pathway differences between cluster 1 and cluster 2. [**(A)**: inositol phosphate metabolism, **(B)**: adipocytokine signaling pathway, **(C)**: endocytosis, **(D)**: phosphatidylinositol signaling system, **(E)**: TGFβ signaling pathway, **(F)**: renal cell carcinoma, **(G)**: mTOR signaling pathway, **(H)**: vasopressin regulated water reabsorption, **(I)**: fatty acid metabolism, **(J)**: leukocyte transendothelial migration, **(K)**: focal adhesion]. NES: normalized enrichment score.

To identify genes related to mast cells, WGCNA was used. The genes were clustered into eight modules (Figure 1D). As determined by Pearson’s correlation coefficients (Figure 1E), the brown module was most highly correlated with mast cell abundance (*r*: 0.58, p = 9e-46). A functional enrichment analysis showed that the genes in the brown module are enriched for the following GO terms: regulation of epithelial cell proliferation, epithelial cell proliferation, regulation of chemotaxis, receptor ligand activity, and signaling receptor activator activity (Figure 1F). A KEGG pathway analysis showed that the mast cell-related genes were involved in the MAPK signaling pathway, Rap 1 signaling pathway, cytokine–cytokine receptor interaction, and PI3K-Akt signaling pathway (Figure 1G). Furthermore, to obtain survival-related mast cell-related genes, we used a univariate Cox regression analysis. Among 250 mast cell-related genes, 103 were related to survival with a threshold of *p* < 0.05 (Supplementary Table S2). An NMF clustering analysis divided these 103 genes in TCGA-KIRC into two molecular subtypes (Cluster 1 and 2) with different molecular and clinical characteristics. Figure 2A shows a heatmap of expression differences between clusters 1 and 2 in the TCGA cohort. The immune score for cluster 2 was significantly higher than that of cluster 1 (*p* < 0.05), with no significant differences in the stromal score and tumor purity between the two subgroups (Figures 2B–D). We also divided ArrayExpress-KIRC and ICGC-KIRC data into cluster 1 and cluster 2. Figure 3A shows a heatmap for the two subgroups in the ArrayExpress cohort. The stromal score for the cluster 1 subgroup was higher than that for the cluster 2 subgroup (Figure 3B). The tumor purity was lower for the cluster 1 subgroup than the cluster 2 subgroup (Figure 3C). There was no significant difference in immune scores between the two subgroups (Figure 3D). Figure 4A shows a heatmap for the two subgroups in the ICGC cohort; the immune score, stromal score, and tumor purity did not differ significantly between cluster 1 and cluster 2 (Figures 4B–D). Differences in the tumor immune microenvironment were observed between the two molecular subtypes in the three KIRC cohorts (Figures 5A–C). As determined by a Kaplan-Meier analysis, survival time was longer in cluster 1 than in cluster 2 (TCGA, *p* < 0.001; ArrayExpress cohort, *p* < 0.001) (Figures 5D–F). A GSEA showed that cluster 1 is enriched for the upregulation of inositol phosphate metabolism, adipocytokine signaling pathway, endocytosis, phosphatidylinositol signaling system, TGFβ signaling pathway, renal cell carcinoma, mTOR signaling pathway, vasopressin regulated water reabsorption, fatty acid metabolism, leukocyte transendothelial migration, and focal adhesion (Figure 6).

### Cluster 2 is More Sensitive to Immuno- and Chemotherapies

**FIGURE 7:**
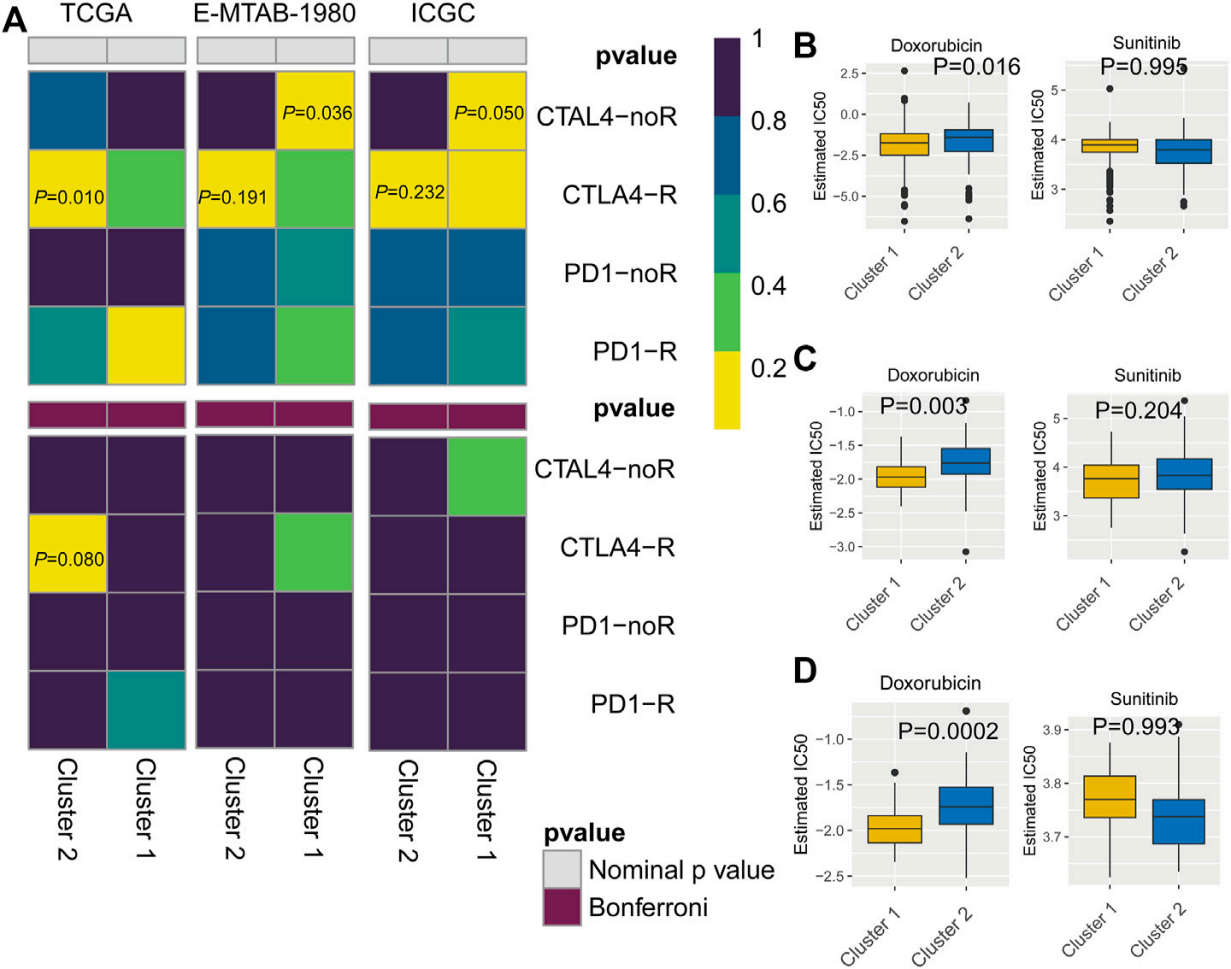
**(A)** Subclass mapping analysis showed that cluster 2 is sensitive to CTLA4-R. [The Cancer Genome Atlas (TCGA): PCTLA4-R = 0.010; E-MTAB-1980: PCTLA4-R = 0.191; International Cancer Genome Consortium (ICGC): PCTLA4-R = 0.232] Based on corrected *p*-values, cluster 1 is not sensitive to CTLA4-R. **(B)** Box plot of estimated IC_50_ values for sunitinib and doxorubicin in cluster 1 and cluster 2. [**(A)**: TCGA; **(B)**: E-MTAB-1980; **(C)**: International Cancer Genome Consortium (ICGC)].

To predict the response to immunotherapy, subclass mapping was applied to compare the expression profiles of the two KIRC subtypes with a published dataset for patients with melanoma treated by immunotherapy (Ro et al., 2017). In the TCGA cohort, cluster 2 was more likely to respond to anti-CTLA4 treatment (*p* = 0.010). However, based on corrected *p*-values, cluster 2 was not sensitive to CTLA4-R (Figure 7A). We also used GDSC data to predict the IC_50_ of doxorubicin for cluster 1 and cluster 2 in the three cohorts. Sensitivity to doxorubicin was significantly higher for cluster 2 than for cluster 1 (TCGA cohort, *p* = 0.016; ArrayExpress cohort, *p* = 0.003; ICGC cohort, *p* = 0.0002) (Figures 7B–D).

### Construction of a Clinical Prognostic Model Based on Mast Cell-Related Genes

Based on 103 mast cell-related genes, we used the LASSO Cox regression algorithm to identify 46 genes with high prognostic value using *p* < 0.01 as a threshold (Supplementary Table S3). Finally, by a multivariate Cox regression analysis, we identified eight genes for the construction of a clinical prognostic model of mast cell-related genes (Table 2). The risk score was calculated as follows: [TRPC4AP expression level × (0.0286)] + [TEK expression level × (−0.0636)] + [IL17RD expression level × (−0.1686)] + [PTH expression level × (1.4582)] + [PDIA2 expression level × (0.1083)] + [SOCS3 expression level × (0.0053)] + [FCGRT expression level × (−0.0117)] + [GDF5 expression level × (0.8070)].

**TABLE 2:** Multivariate Cox regression analysis of genes related to mast cells used to construct the model.

Gene	Coefficient	HR	HR.95 L	HR.95H	*p*-value
TRPC4AP	0.02857631	1.02898853	1.00929749	1.04906374	0.00374682
TEK	−0.0635723	0.93840629	0.90164062	0.97667113	0.00182356
IL17RD	−0.1686320	0.84481971	0.75159926	0.94960225	0.00470115
PTH	1.45824552	4.29841145	0.82061110	22.5153437	0.08435432
PDIA2	0.10826512	1.11434314	0.99652289	1.24609344	0.05758127
SOCS3	0.00534328	1.00535758	1.00240904	1.00831479	0.00036303
FCGRT	−0.0117308	0.98833770	0.97729089	0.99950937	0.04080188
GDF5	0.80699657	2.24116669	1.40590172	3.57267371	0.00069420

### Application of the Prognostic Model to Patients With KIRC

**FIGURE 8:**
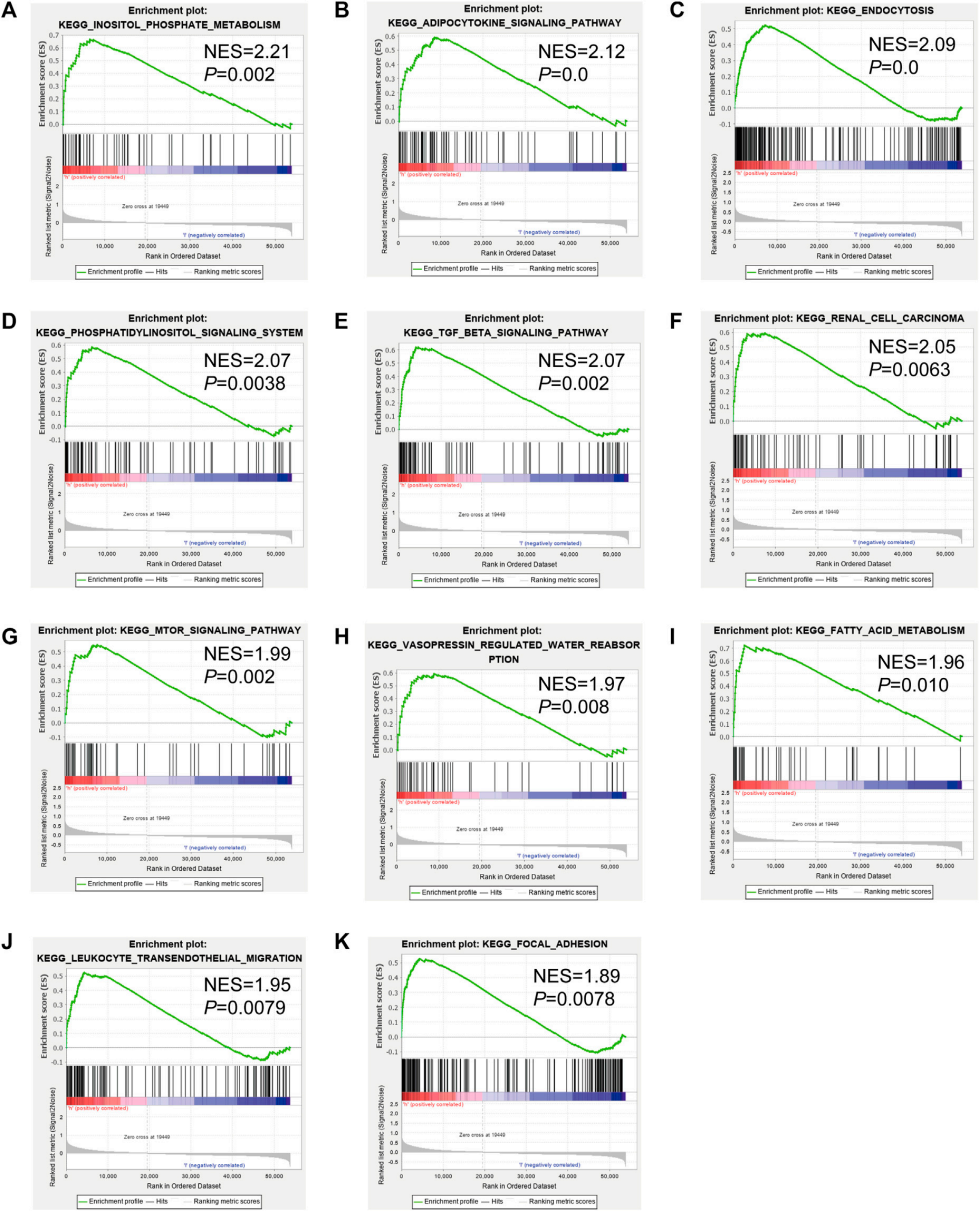
**(A–E)** Distribution of patients according to the risk index. **(F–J)** Risk score calculated from the clinical prognostic model can predict survival. **(K–O)** Receiver operating characteristic (ROC) curve to verify the prognostic value of the model. [**(A, F, K)**: The Cancer Genome Atlas (TCGA); **(B, G, L)**: TCGA training group; **(C, H, M)**: TCGA testing group; **(D, I, N)**: E-MTAB-1980; **(E, J, O)**: International Cancer Genome Consortium (ICGC)].

At a ratio of 1:1, patients with KIRC in the TCGA cohort were divided into training and test sets. Based on the risk score calculated from the clinical prognostic model based on mast cell-related genes, patients with KIRC were divided into high-risk and low-risk groups (Figure 8B). As the risk score increased, the survival time of patients with KIRC decreased (Figure 8G). A time-dependent ROC curve analysis supported the predictive value of the model. These results show that our prognostic indicators have a good performance (Figure 8L).

### Validation of the Clinical Prognostic Model

To determine the reliability of the clinical prognostic model across populations, we applied the formula to the TCGA cohort, TCGA testing cohort, ArrayExpress cohort, and ICGC cohort, yielding similar results to those obtained for the training set. Patients with KIRC were divided into high-risk or low-risk groups based on the risk score calculated from the model (Figure 8A, C–E). A lower risk was associated with a longer survival time (Figure 8B, H–J). Additionally, we verified the predictive accuracy of the clinical prognostic model in a joint analysis of the TCGA cohort, TCGA testing group, ArrayExpress cohort, and ICGC cohort (Figure 8C, M–O). Therefore, the newly developed clinical prognostic model is generalizable to different populations.

### Survival Analysis and Methylation Analysis of Eight Genes Included in the Prognostic Model

**FIGURE 9:**
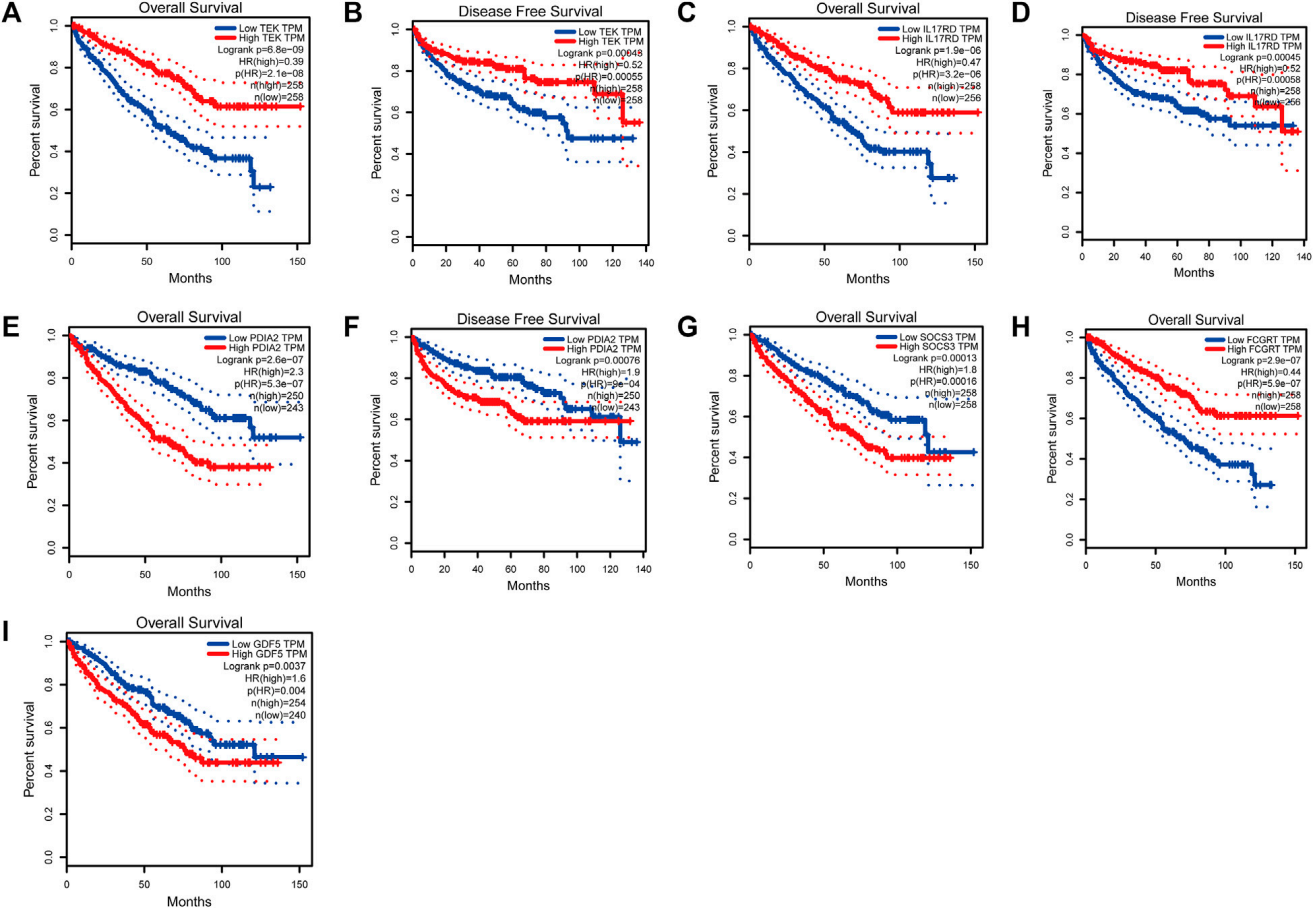
Analysis of overall survival (OS) and disease-free survival (DFS) by the application of the constructed model based on mast cell-related genes of patients with kidney renal clear cell carcinoma (KIRC). (TEK: p_OS_ < 0.001, p_DFS_ = 0.00043; IL17RD: p_OS_ < 0.001, p_DFS_ = 0.00045; FCGRT: p_OS_ < 0.001; PDIA2: p_OS_ < 0.001, p_DFS_ = 0.00076; SOCS3: p_OS_ = 0.00013; GDF5: p_OS_ = 0.00013).

**FIGURE 10:**
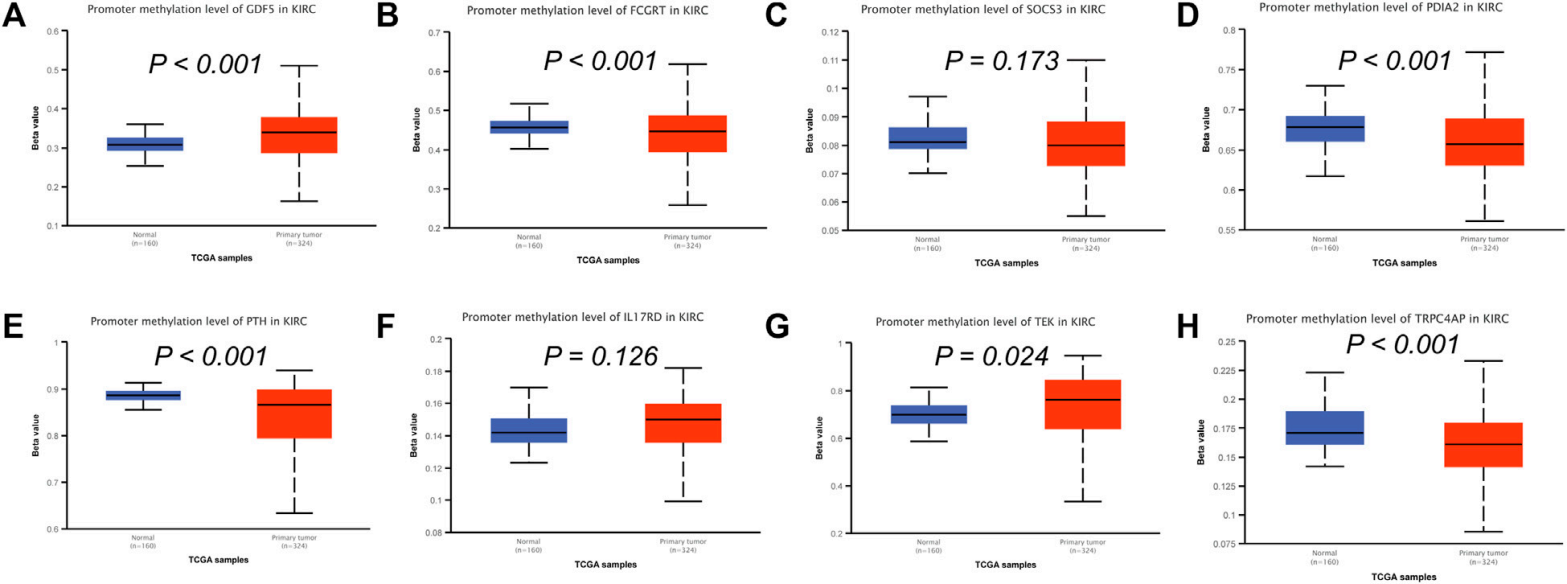
Analysis of promoter methylation levels of mast cell-related genes in the constructed model. **(A–H)** GDF5: *p* < 0.001; SOCS3: *p* = 0.173; FCGRT: *p* < 0.001; PDIA2: *p* < 0.001; PTH: *p* < 0.001; IL17RD: *p* = 0.126; TEK: *p* = 0.024; TRPC4AP: *p* < 0.001.

To analyze the correlation between OS in patients with KIRC and the expression of the eight mast cell genes included in the model, the GEPIA database was utilized. OS was better for patients with low TEK expression than with high TEK expression (*p* < 0.001). Patients with high TEK expression had better disease-free survival (DFS) than that of patients with low TEK expression (*p* = 0.00043). Patients with high IL17RD expression had better OS than that of patients with low IL17RD expression (*p* < 0.001). Patients with high IL17RD expression had better OS than that of patients with low IL17RD expression (*p* = 0.00045). Patients with high FCGRT expression had better OS than that of patients with low FCGRT expression (*p* < 0.001). In contrast, patients with low PDIA2 expression had better OS than that of patients with high PDIA2 expression (*p* < 0.001). Patients with low PDIA2 expression had a better DFS than that of patients with high PDIA2 expression (*p* < 0.001). Patients with low SOCS3 expression had better OS than that of patients with high SOCS3 expression (*p* = 0.00013). Patients with low GDF5 expression had better OS than that of patients with high GDF5 expression (*p* = 0.00013) (Figure 9). In addition, we analyzed the promoter methylation levels of eight genes using the UALCAN database. The promoter methylation levels of FCGRT (*p* < 0.001), PDIA2 (*p* < 0.001), PTH (*p* < 0.001), and TRPC4AP (*p* < 0.001) were decreased in KIRC and those of GDF5 (*p* < 0.001) and TEK (*p* = 0.024) were increased in KIRC (Figure 10).

## Discussion

KIRC is the third most common malignant tumor of the urinary system. In 2020, it accounted for an estimated 14,830 deaths, with approximately 73,750 new cases in the United States (Siegel et al., 2020). Surgery remains the best treatment option. However, most patients eventually develop distant metastases (Rao et al., 2018). At present, radiotherapy, chemotherapy, targeted therapy, and immunotherapy are not effective in KIRC (Lalani et al., 2018). Therefore, a deeper understanding of the molecular mechanisms underlying KIRC is necessary for development of effective early diagnostic methods and prognostic markers.

Mast cells are important components of the immune microenvironment of tumor tissues and can promote or inhibit tumorigenesis by releasing various factors (Varricchi et al., 2017). We quantified the abundance of mast cells in three cohorts and found that the mast cell gene set used in the study is a protective factor in KIRC. We identified mast cell genes that were most closely related to the ssGSEA score by WGCNA. A functional enrichment analysis showed that mast cell-related genes are related to epithelial cell proliferation regulation, epithelial cell proliferation, chemotaxis regulation, receptor ligand activity, signal receptor activator activity, MAPK signaling pathway, Rap1 signaling pathway, cytokine receptor interaction, and PI3K/Akt signaling pathway. Furthermore, after screening for survival-related mast cell-related genes, we divided KIRC into two molecular subtypes, cluster 1 and cluster 2, based on these genes. Predictive analyses of the responses to immuno-chemotherapies indicated that cluster 2 is sensitive to anti-CTLA4 treatment based on the *p*-value but not based on the corrected *p*-value. The mechanism underlying the observed difference in sensitivity requires further research. In addition, sensitivity to doxorubicin was higher for cluster 2 than cluster 1.

Finally, we constructed a clinical prognostic model based on mast cell-related genes using the LASSO Cox regression model and multivariate Cox regression model to predict the prognosis and survival time of patients with KIRC. Time-dependent ROC supported the predictive ability of the model.

The clinical prognostic model was based on eight mast cell-related genes. The tyrosine kinase receptor TEK, mainly expressed on endothelial cells, is activated by Angiopoietin-1. Endothelial cell survival and vascular maturation are promoted by the activation and phosphorylation of TEK, leading to downstream signal transduction (Eklund and Saharinen, 2013). Moreover, TEK promotes immune responses, the activation of mast cells, and the adhesion of mast cells to VCAM-1 (Kanemaru et al., 2015). Low TEK expression promotes AKT phosphorylation, the epithelial–mesenchymal transition, and the proliferation and migration of KIRC cells and inhibits the apoptosis of KIRC cells (Chen et al., 2021). In addition, the mitogen-activated protein kinase (MAPK) pathway is related to senescence, apoptosis, cell proliferation, differentiation, and migration (Sun et al., 2015). Cytokine signal transduction 3 (SOCS3) is an inhibitor of IL-6 and a negative regulator of cytokine signal transduction. SOCS3 not only inhibits cytokine-mediated JAK/STAT signal transduction, but also maintains the MAPK pathway, thereby promoting the growth of KIRC and angiogenesis (Oguro et al., 2013). Very few studies of KIRC have focused on TRPC4AP, IL17RD, PTH, PDIA2, FCGRT, and GDF5, and the role of these mast cell-related genes in KIRC requires further research.

Epigenetic changes often occur in KIRC and may be important events in its development (Joosten et al., 2018). Abnormal DNA methylation is a common type of epigenetic change, including genome-wide changes and regional variation (Jones and Baylin, 2002; Feinberg and Tycko, 2004). Abnormal DNA methylation can induce the abnormal expression of cancer-related genes and is the most common epigenetic change in tumorigenesis. Changes in DNA methylation during tumor progression affect target tumor cells; additionally, the immune system may undergo methylation changes during immune responses (Li et al., 2017). In our study, the promoter methylation levels of FCGRT, PDIA2, PTH, and TRPC4AP were reduced in KIRC. In contrast, promoter methylation levels of GDF5 and TEK were elevated in KIRC. We believe that a decrease in TEK resulting from an increase in promoter methylation levels may promote the proliferation and migration of KIRC cells, ultimately leading to the occurrence and progression of KIRC. Of course, the mechanism underlying the changes in the methylation levels of these genes in KIRC needs further verification.

The prognostic value of the newly established model was supported by an analysis of the OS of patients with KIRC in a training group, in which patients classified as high-risk had a shorter survival time. In addition, we used the risk scores calculated from the prognosis model to generate a risk curve to monitor disease progression. The ROC curve showed that our clinical prognostic model had a high predictive value. All results were verified by ArrayExpress and ICGC cohorts. Therefore, this model may be valuable for evaluating the prognosis of patients with KIRC.

This study had some limitations. First, all data were collected from TCGA, ArrayExpress, and ICGC, but lack of a support from hospital centre. Second, experimental studies of the functions of mast cell-related genes were not conducted. Therefore, further verification is needed to clarify the molecular mechanisms underlying KIRC and the roles of mast cells.

## Conclusion

We found a correlation between prognosis and mast cell abundance in KIRC. By WGCNA, genes related to mast cells were identified, and two molecular subtypes (cluster 1 and cluster 2) were identified. Patients in cluster 2 were more likely to benefit from immunotherapy. The newly developed clinical prognostic model based on eight mast cell-related genes may contribute to the monitoring and the prediction of survival. More broadly, our research provides a basis for personalized medicine in KIRC.

## Data Availability

The original contributions presented in the study are included in the article/Supplementary Material, further inquiries can be directed to the corresponding author.
