# Supplementary material for: Identification of Mast Cell-Based Molecular Subtypes and a Predictive Signature in Clear Cell Renal Cell Carcinoma
Source: Front Mol Biosci. 2021 Sep 27;8:719982. doi: 10.3389/fmolb.2021.719982 (PMC8503328; doi:10.3389/fmolb.2021.719982)
Supplement: Supplementary file 1 [file Table1.DOCX]

Supplementary Table 1. The mast cell gene set was used in this study.

| Gene name |
| --- |
| PRG2 |
| CTSG |
| SLC18A2 |
| CPA3 |
| TPSB2 |
| MS4A2 |
| TPSAB1 |
| GATA2 |
| HDC |
| ELA2 |
| LOH11CR2A |
| CMA1 |
| PGDS |
| MLPH |
| ADCYAP1 |
| SIGLEC6 |
| CALB2 |
| SLC24A3 |
| KIT |
| TAL1 |
| ABCC4 |
| PPM1H |
| MAOB |
| HPGD |
| SCG2 |
| PTGS1 |
| CEACAM8 |
| MPO |
| NR0B1 |
| LOC339524 |

Supplement Table 2. 103 Mast cell-related genes related to survival.

| id | HR | HR.95L | HR.95H | pvalue |
| --- | --- | --- | --- | --- |
| TEK | 0.89551 | 0.864454 | 0.927682 | 8.88E-10 |
| TRPC4AP | 1.050845 | 1.032201 | 1.069826 | 5.64E-08 |
| PDGFD | 0.968117 | 0.95682 | 0.979548 | 6.29E-08 |
| EDNRB | 0.981131 | 0.974214 | 0.988097 | 1.31E-07 |
| KDR | 0.982867 | 0.976559 | 0.989216 | 1.43E-07 |
| CX3CL1 | 0.978334 | 0.970328 | 0.986407 | 1.75E-07 |
| HSPA1L | 0.419597 | 0.301943 | 0.583096 | 2.31E-07 |
| S1PR1 | 0.975965 | 0.966948 | 0.985067 | 2.80E-07 |
| SEMA3G | 0.895697 | 0.858368 | 0.934649 | 3.94E-07 |
| TGFBR2 | 0.987811 | 0.982907 | 0.99274 | 1.37E-06 |
| PLXNA2 | 0.85736 | 0.805195 | 0.912906 | 1.55E-06 |
| GDF5 | 2.6573 | 1.775841 | 3.976281 | 2.01E-06 |
| AKT3 | 0.907893 | 0.872308 | 0.94493 | 2.17E-06 |
| RARB | 0.841388 | 0.783067 | 0.904053 | 2.45E-06 |
| THRA | 0.908412 | 0.872685 | 0.945602 | 2.70E-06 |
| IL17RD | 0.723241 | 0.630703 | 0.829357 | 3.51E-06 |
| TGFBR3 | 0.855912 | 0.801407 | 0.914124 | 3.58E-06 |
| PIK3R3 | 0.926998 | 0.897676 | 0.957278 | 3.79E-06 |
| PLXNB3 | 1.205579 | 1.112736 | 1.30617 | 4.82E-06 |
| TCF7L2 | 0.828944 | 0.764464 | 0.898862 | 5.61E-06 |
| CALCRL | 0.967343 | 0.953517 | 0.98137 | 6.18E-06 |
| INSR | 0.979289 | 0.970425 | 0.988233 | 6.43E-06 |
| FLT1 | 0.985619 | 0.979397 | 0.991882 | 7.38E-06 |
| MAPT | 0.920579 | 0.887673 | 0.954705 | 8.36E-06 |
| A2M | 0.996978 | 0.995636 | 0.998323 | 1.08E-05 |
| PSMD14 | 1.134287 | 1.070584 | 1.201781 | 1.93E-05 |
| PLTP | 1.002368 | 1.001252 | 1.003484 | 3.13E-05 |
| PTK2 | 0.885519 | 0.834986 | 0.93911 | 5.00E-05 |
| NRP1 | 0.981834 | 0.973025 | 0.990722 | 6.69E-05 |
| JAG1 | 0.974807 | 0.962419 | 0.987355 | 9.22E-05 |
| CSPG5 | 3.485778 | 1.856033 | 6.546569 | 0.000103 |
| AGTR1 | 0.922065 | 0.88498 | 0.960704 | 0.000107 |
| GDF6 | 0.919791 | 0.881518 | 0.959727 | 0.000115 |
| ACVRL1 | 0.956727 | 0.935315 | 0.97863 | 0.000128 |
| LIF | 1.012479 | 1.00605 | 1.01895 | 0.000136 |
| IL20RB | 1.007712 | 1.00371 | 1.01173 | 0.000154 |
| PDGFB | 0.964293 | 0.946251 | 0.982679 | 0.000161 |
| FCGRT | 0.979706 | 0.969189 | 0.990337 | 0.000197 |
| PDIA2 | 1.193846 | 1.08726 | 1.31088 | 0.000205 |
| PDGFRL | 1.038168 | 1.017655 | 1.059095 | 0.000234 |
| MAPK3 | 0.957579 | 0.934459 | 0.981271 | 0.000509 |
| APLNR | 0.980297 | 0.969243 | 0.991478 | 0.000583 |
| ARRB1 | 0.8642 | 0.795119 | 0.939283 | 0.000596 |
| SOCS3 | 1.004272 | 1.001822 | 1.006727 | 0.000623 |
| RASGRP3 | 0.865698 | 0.795693 | 0.941863 | 0.000802 |
| PTH | 10.15502 | 2.613038 | 39.46532 | 0.000817 |
| AQP9 | 1.017378 | 1.006706 | 1.028162 | 0.001363 |
| ADIPOQ | 1.14355 | 1.052856 | 1.242056 | 0.001464 |
| SORT1 | 0.95758 | 0.932156 | 0.983698 | 0.001593 |
| CYSLTR1 | 0.633998 | 0.477116 | 0.842466 | 0.001679 |
| GDF7 | 0.627051 | 0.466727 | 0.842449 | 0.001949 |
| TGFB1 | 1.008111 | 1.002944 | 1.013304 | 0.002059 |
| CTSG | 0.726986 | 0.592366 | 0.8922 | 0.002276 |
| NR2F1 | 0.964572 | 0.942478 | 0.987185 | 0.002281 |
| ENG | 0.995613 | 0.992768 | 0.998466 | 0.002597 |
| FYN | 0.94702 | 0.9135 | 0.981771 | 0.003071 |
| RAC3 | 1.067385 | 1.021827 | 1.114974 | 0.003388 |
| CMA1 | 0.307926 | 0.139461 | 0.679893 | 0.003561 |
| PDGFRA | 1.054187 | 1.017211 | 1.092507 | 0.003772 |
| FLT4 | 0.959539 | 0.933077 | 0.986752 | 0.003796 |
| CBL | 0.872314 | 0.793101 | 0.959439 | 0.004917 |
| CMTM8 | 0.873299 | 0.793613 | 0.960987 | 0.005518 |
| RAET1E | 0.145634 | 0.037203 | 0.570088 | 0.005657 |
| LPA | 0.005728 | 0.000147 | 0.222892 | 0.005718 |
| EDN1 | 0.990264 | 0.983392 | 0.997185 | 0.005899 |
| PTHLH | 1.005479 | 1.001573 | 1.0094 | 0.005928 |
| SEMA3B | 1.023556 | 1.006714 | 1.04068 | 0.005952 |
| TGFB3 | 1.02684 | 1.007362 | 1.046694 | 0.006714 |
| BMP8A | 1.362109 | 1.08705 | 1.706768 | 0.007248 |
| ROBO2 | 0.296717 | 0.119556 | 0.736399 | 0.0088 |
| RBP7 | 0.991932 | 0.985926 | 0.997975 | 0.008943 |
| ESM1 | 0.99682 | 0.994439 | 0.999208 | 0.009067 |
| SEMA3F | 0.979856 | 0.964975 | 0.994966 | 0.00915 |
| TIE1 | 0.969938 | 0.947906 | 0.992482 | 0.009222 |
| NFATC4 | 1.082724 | 1.016566 | 1.153188 | 0.013485 |
| BMP4 | 0.946624 | 0.906244 | 0.988803 | 0.013655 |
| BMP5 | 0.660654 | 0.475064 | 0.918747 | 0.013754 |
| LBP | 1.001006 | 1.000199 | 1.001813 | 0.014524 |
| VIPR1 | 0.543767 | 0.330877 | 0.893632 | 0.016231 |
| TACR1 | 0.507805 | 0.291827 | 0.883628 | 0.016498 |
| BMP6 | 0.913784 | 0.84883 | 0.983709 | 0.01655 |
| TPM2 | 1.003374 | 1.000611 | 1.006145 | 0.016675 |
| ARTN | 1.089732 | 1.015151 | 1.169792 | 0.017517 |
| IL4R | 1.030012 | 1.005114 | 1.055527 | 0.017858 |
| ESR2 | 1.800836 | 1.104045 | 2.937389 | 0.01845 |
| TNFRSF4 | 1.035316 | 1.005814 | 1.065683 | 0.018623 |
| VEGFC | 1.039249 | 1.006305 | 1.073273 | 0.019163 |
| RXRA | 0.945443 | 0.90186 | 0.991133 | 0.019814 |
| NMBR | 0.011574 | 0.00026 | 0.514546 | 0.021269 |
| NMB | 1.002946 | 1.000366 | 1.005533 | 0.025195 |
| HDAC1 | 1.02979 | 1.003213 | 1.057071 | 0.027774 |
| CLEC11A | 1.01394 | 1.001458 | 1.026577 | 0.028485 |
| APOD | 1.019134 | 1.00192 | 1.036643 | 0.029205 |
| PGF | 1.002806 | 1.000267 | 1.005351 | 0.030306 |
| JAG2 | 0.957885 | 0.921252 | 0.995974 | 0.030562 |
| TMEM173 | 1.019253 | 1.001539 | 1.037279 | 0.033014 |
| GNAI1 | 0.961676 | 0.927718 | 0.996876 | 0.033126 |
| FGF1 | 0.860337 | 0.746766 | 0.99118 | 0.037287 |
| PF4V1 | 1.014327 | 1.000558 | 1.028285 | 0.041351 |
| PNOC | 1.063552 | 1.001735 | 1.129183 | 0.043728 |
| PLXNA4 | 1.153546 | 1.003543 | 1.32597 | 0.04446 |
| SSTR5 | 1.526544 | 1.00692 | 2.314322 | 0.046322 |
| MCHR1 | 1.012763 | 1.000095 | 1.025591 | 0.048299 |

Supplement Table 3. 46 Mast cell-related genes with survival prognostic value.

| id | HR | HR.95L | HR.95H | pvalue |
| --- | --- | --- | --- | --- |
| RASGRP3 | 0.865698 | 0.795693 | 0.941863 | 0.000802 |
| TCF7L2 | 0.828944 | 0.764464 | 0.898862 | 5.61E-06 |
| ACVRL1 | 0.956727 | 0.935315 | 0.97863 | 0.000128 |
| TRPC4AP | 1.050845 | 1.032201 | 1.069826 | 5.64E-08 |
| ARRB1 | 0.8642 | 0.795119 | 0.939283 | 0.000596 |
| EDNRB | 0.981131 | 0.974214 | 0.988097 | 1.31E-07 |
| SEMA3G | 0.895697 | 0.858368 | 0.934649 | 3.94E-07 |
| IL20RB | 1.007712 | 1.00371 | 1.01173 | 0.000154 |
| AKT3 | 0.907893 | 0.872308 | 0.94493 | 2.17E-06 |
| PIK3R3 | 0.926998 | 0.897676 | 0.957278 | 3.79E-06 |
| PDGFB | 0.964293 | 0.946251 | 0.982679 | 0.000161 |
| LIF | 1.012479 | 1.00605 | 1.01895 | 0.000136 |
| TEK | 0.89551 | 0.864454 | 0.927682 | 8.88E-10 |
| IL17RD | 0.723241 | 0.630703 | 0.829357 | 3.51E-06 |
| PTH | 10.15502 | 2.613038 | 39.46532 | 0.000817 |
| CX3CL1 | 0.978334 | 0.970328 | 0.986407 | 1.75E-07 |
| NRP1 | 0.981834 | 0.973025 | 0.990722 | 6.69E-05 |
| PTK2 | 0.885519 | 0.834986 | 0.93911 | 5.00E-05 |
| JAG1 | 0.974807 | 0.962419 | 0.987355 | 9.22E-05 |
| KDR | 0.982867 | 0.976559 | 0.989216 | 1.43E-07 |
| A2M | 0.996978 | 0.995636 | 0.998323 | 1.08E-05 |
| PLXNA2 | 0.85736 | 0.805195 | 0.912906 | 1.55E-06 |
| PLTP | 1.002368 | 1.001252 | 1.003484 | 3.13E-05 |
| CALCRL | 0.967343 | 0.953517 | 0.98137 | 6.18E-06 |
| MAPT | 0.920579 | 0.887673 | 0.954705 | 8.36E-06 |
| PLXNB3 | 1.205579 | 1.112736 | 1.30617 | 4.82E-06 |
| PDIA2 | 1.193846 | 1.08726 | 1.31088 | 0.000205 |
| S1PR1 | 0.975965 | 0.966948 | 0.985067 | 2.80E-07 |
| RARB | 0.841388 | 0.783067 | 0.904053 | 2.45E-06 |
| FLT1 | 0.985619 | 0.979397 | 0.991882 | 7.38E-06 |
| TGFBR3 | 0.855912 | 0.801407 | 0.914124 | 3.58E-06 |
| SOCS3 | 1.004272 | 1.001822 | 1.006727 | 0.000623 |
| GDF6 | 0.919791 | 0.881518 | 0.959727 | 0.000115 |
| PDGFD | 0.968117 | 0.95682 | 0.979548 | 6.29E-08 |
| FCGRT | 0.979706 | 0.969189 | 0.990337 | 0.000197 |
| CSPG5 | 3.485778 | 1.856033 | 6.546569 | 0.000103 |
| PSMD14 | 1.134287 | 1.070584 | 1.201781 | 1.93E-05 |
| PDGFRL | 1.038168 | 1.017655 | 1.059095 | 0.000234 |
| GDF5 | 2.6573 | 1.775841 | 3.976281 | 2.01E-06 |
| AGTR1 | 0.922065 | 0.88498 | 0.960704 | 0.000107 |
| MAPK3 | 0.957579 | 0.934459 | 0.981271 | 0.000509 |
| TGFBR2 | 0.987811 | 0.982907 | 0.99274 | 1.37E-06 |
| APLNR | 0.980297 | 0.969243 | 0.991478 | 0.000583 |
| HSPA1L | 0.419597 | 0.301943 | 0.583096 | 2.31E-07 |
| THRA | 0.908412 | 0.872685 | 0.945602 | 2.70E-06 |
| INSR | 0.979289 | 0.970425 | 0.988233 | 6.43E-06 |
