# Supplementary figures and images for: Identification of Mast Cell-Based Molecular Subtypes and a Predictive Signature in Clear Cell Renal Cell Carcinoma
Source: Front Mol Biosci. 2021 Sep 27;8:719982. doi: 10.3389/fmolb.2021.719982 (PMC8503328; doi:10.3389/fmolb.2021.719982)

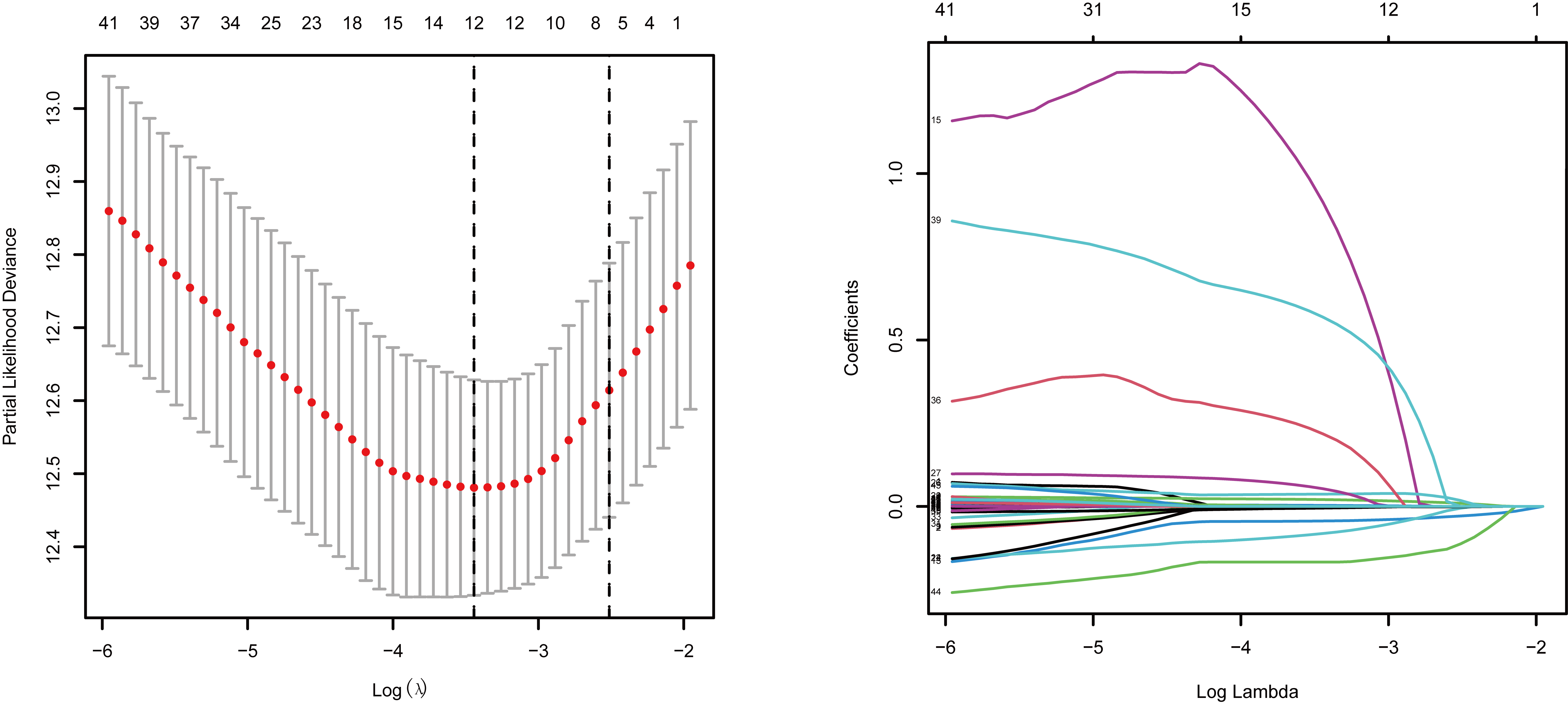

Supplement: Supplementary file 2 [file Image1.TIF]
